# Exploring the Landscape of Standards and Guidelines in AgeTech Design and Development: Scoping Review and Thematic Analysis

**DOI:** 10.2196/58196

**Published:** 2024-10-31

**Authors:** Shahabeddin Abhari, Josephine McMurray, Tanveer Randhawa, Gaya Bin Noon, Thokozani Hanjahanja-Phiri, Heather McNeil, Fiona Manning, Patricia Debergue, Jennifer Teague, Plinio Pelegrini Morita

**Affiliations:** 1 School of Public Health Sciences, University of Waterloo Waterloo, ON Canada; 2 Lazaridis School of Business & Economics/Community Health, Wilfrid Laurier University Waterloo, ON Canada; 3 National Research Council Canada, Aging in Place Challenge Program Ottawa, ON Canada; 4 Canadian Standards Association Toronto, ON Canada; 5 Department of Systems Design Engineering, University of Waterloo Waterloo, ON Canada; 6 Research Institute for Aging, University of Waterloo Waterloo, ON Canada; 7 Centre for Digital Therapeutics, Techna Institute, University Health Network Toronto, ON Canada; 8 Dalla Lana School of Public Health, Institute of Health Policy, Management, and Evaluation, University of Toronto Toronto, ON Canada

**Keywords:** aging in place, technology, gerontechnology, AgeTech, assistive technology, older adult, aging, ambient assisted living, active assisted living

## Abstract

**Background:**

AgeTech (technology for older people) offers digital solutions for older adults supporting aging in place, including digital health, assistive technology, Internet of Things, medical devices, robotics, wearables, and sensors. This study underscores the critical role of standards and guidelines in ensuring the safety and effectiveness of these technologies for the health of older adults. As the aging demographic expands, the focus on robust standards becomes vital, reflecting a collective commitment to improving the overall quality of life for older individuals through thoughtful and secure technology integration.

**Objective:**

This scoping review aims to investigate the current state of standards and guidelines applied in AgeTech design and development as reported in academic literature. We explore the existing knowledge of these standards and guidelines and identify key gaps in the design and development of AgeTech guidelines and standards in scholarly publications.

**Methods:**

The literature review adhered to the PRISMA-ScR (Preferred Reporting Items for Systematic Reviews and Meta-Analyses Extension for Scoping Reviews) guidelines. Searches were carried out across multiple databases, including Scopus, IEEE, PubMed, Web of Science, EBSCO, CINAHL, Cochrane, and Google Scholar, using a search string incorporating concepts such as “older people,” “technology,” and “standards or guidelines.” Alternative terms, Boolean operators, and truncation were used for comprehensive coverage in each database. The synthesis of results and data analysis involved both quantitative and qualitative methods.

**Results:**

Initially, 736 documents were identified across various databases. After applying specific inclusion and exclusion criteria and a screening process, 58 documents were selected for full-text review. The findings highlight that the most frequently addressed aspect of AgeTech standards or guidelines is related to “design and development,” constituting 36% (21/58) of the literature; “usability and user experience” was the second most prevalent aspect, accounting for 19% (11/58) of the documents. In contrast, “privacy and security” (1/58, 2%) and “data quality” (1/58, 2%) were the least addressed aspects. Similarly, “ethics,” “integration and interoperability,” “accessibility,” and “acceptance or adoption” each accounted for 3% (2/58) of the documents. In addition, a thematic analysis identified qualitative themes that warrant further exploration of variables.

**Conclusions:**

This study investigated the available knowledge regarding standards and guidelines in AgeTech design and development to evaluate their current status in academic literature. The substantial focus on assistive technologies and ambient assisted living technologies confirmed their vital role in AgeTech. The findings provide valuable insights for interested parties and point to prioritized areas for further development and research in the AgeTech domain.

## Introduction

### Background

As the global population ages, the number of people aged ≥60 years is expected to double, reaching 2.1 billion by 2050, while the number of those aged ≥80 years is expected to triple, reaching 426 million [1-3]. This trend is mirrored in Canada, where older adults are projected to make up approximately 25% of the population by 2050 [4,5]. Notably, >95% of older Canadians would prefer to age in their own homes and communities [6,7]. However, many older adults contend with multiple chronic health conditions that can necessitate long-term care to manage their activities of daily living. A recent Canadian study suggests that between 11% and 22% of older adults transitioning into long-term care could have remained in their homes or community-based care settings with the appropriate supports in place [8].

Addressing the needs of this rapidly aging population requires innovative solutions to overcome the unique social, fiscal, and medical challenges of providing health and social care. Assistive technologies, including a range of devices, equipment, software, or adaptations to the physical environment, offer the potential to improve functional ability, social participation, and well-being, making them a valuable part of the solution. Within this broad category, AgeTech—technology for older people, a specialized subset of assistive technologies—is designed to meet the unique needs and preferences of older adults and their care partners, focusing on preserving and enhancing independence and inclusion for individuals aged ≥65 years [9-11]. For the purposes of this study, a broad definition of AgeTech is used [9]: “AgeTech refers to digital technologies or digitally enabled products designed explicitly for or with the potential to provide benefit to older adults and care partners. This will include a range of innovations supporting aging in place, healthy aging, staying connected, and more. It is expected that technologies in the program will include digital health, assistive technologies, Internet of Things, medical devices/diagnostics, robotics, wearables, and other sensor-based technologies.”

Despite the potential of AgeTech to help older adults age longer in the homes and communities of their choice, many technologies have not achieved the expected success rates [12]. Challenges include the development of fit-for-purpose technologies that address the real needs of older adults and that are subsequently adopted by them and their care partners [12,13]. Recent studies have revealed evidence of the potential impact of technology in supporting older adults to age in place [9,14]. Still, AgeTech innovators can struggle with the adoption of AgeTech products or services by older people, their caregivers, and the systems that help care for them. One factor contributing to this translational challenge is the lack of standards-based solutions for AgeTech design and evaluation, which can result in products that are either unfit for purpose or fail to address real needs [13,15]. Standards and guidelines are crucial in AgeTech development for ensuing support for safety, efficacy, reliability, interoperability, and regulatory compliance [16,17]. They address the specific needs of older adults, mitigate risks, and foster user trust. In addition, standards promote seamless integration, improving care coordination and decision-making. Consistency and quality across AgeTech solutions are maintained, fostering innovation and reliability. Compliance with standards streamlines regulatory processes and enhances market acceptance, benefiting older adults and caregivers. Overall, standards are essential in advancing AgeTech, enhancing aging-related care, and improving the well-being of older populations [9,15-19].

### Objectives

This scoping review aims to explore the current state of standards and guidelines used in the design and development of AgeTech in the academic peer-reviewed literature and highlights the importance of these frameworks in advancing the field [9,15-19]. The decision to initially focus on academic peer-reviewed literature allows for a rigorous, systematic exploration of the foundational research in AgeTech design and development standards and guidelines. This approach ensures that the review is grounded in scientifically validated findings, providing a robust framework from which to explore the broader, practical implications and innovations captured within the gray literature in a subsequent review. This phased methodology strategically broadens the scope of analysis to include a comprehensive spectrum of insights, from theoretical underpinnings to practical applications in the field. Two research questions helped to guide the search strategy and data extraction process in the academic literature:

1. Research question 1: What is the existing knowledge about standards or guidelines in the design and development of AgeTech?
2. Research question 2: What are the key gaps in the design and development of standards or guidelines for AgeTech?

## Methods

### Study Design

A scoping review was chosen as the systematic method to comprehensively map the available evidence and provide an overview of the scientific literature concerning existing guidelines and standards for the development of AgeTech [20-22]. The scoping review followed a five-step process: (1) identifying an initial research question; (2) identifying relevant studies; (3) selecting the studies; (4) charting the data; and (5) collating, summarizing, and reporting the findings [20]. To establish an effective search strategy and search strings, our research team engaged in 3 consultations with a librarian subject matter expert. Following these consultations and using an iterative approach to test database and search terms, a search strategy was finalized. Article selection adhered to the guidelines outlined in the PRISMA-ScR (Preferred Reporting Items for Systematic Reviews and Meta-Analyses Extension for Scoping Reviews).

### Information Sources and Study Selection

The research team performed the scoping review search in October 2023. A subsequent search was conducted before submission for publication on January 20, 2024. We searched 8 databases, including Scopus, IEEE, PubMed, Web of Science, EBSCO, CINAHL, Cochrane, and Google Scholar, to identify academic, peer-reviewed journal articles and conference papers. Reflective of the interdisciplinary nature of our research, we searched a broad range of databases and knowledge repositories in the health sciences and engineering disciplines. This strategy was designed to ensure a comprehensive review of relevant literature. The retrieval period for the search was not limited. The search strategy, including keywords and search strings, is summarized in Textbox 1.

Articles were imported into Covidence (Veritas Health Innovation Ltd) screening and data extraction software for conducting systematic reviews. All titles and abstracts were screened by at least 2 members of the research team (SA, TR, or JM) using the inclusion and exclusion criteria detailed in Textbox 2. Any discrepancies were reviewed by a member who was not an original reviewer.

Articles identified in the title and abstract screening as relevant were included in a full-text review using the same inclusion and exclusion criteria and review process. To identify any studies that may have been missed during the initial search, we conducted both forward and backward searching.

**Academic peer-reviewed journal search strategy.**

**Search strategy**

- Databases: Scopus, IEEE, PubMed, Web of Science, EBSCO, CINAHL, Cochrane, and Google Scholar
- Limits: language (only resources in English) and species (studies on human)
- Date: all literature till January 20, 2024
- Search string: #1 AND #2 AND #3
  #1 “standards” OR “guideline*”
  #2 “Agetech” OR “ambient assisted living” OR “active assisted living” OR “wearables” OR “mobile digital technology” OR “remote patient monitoring” OR “telemedicine” OR “telehealth” OR “gerontology” OR “digital technology” OR “mhealth” OR “mobile health” OR “assistive technology” OR “internet of things” OR “virtual reality” OR “voice recognition” OR “artificial intelligence” OR “smart technology” OR “smart assistive technology” OR “digital” OR “technology”
  #3 “Aging” OR “ageing” OR “elderly” OR “seniors” OR “aging in place” OR “older adult” OR “care partner” OR “senior citizens” OR “aging population” OR “gerentol*” OR “geriatric” OR “care givers”

**Inclusion and exclusion criteria.**

**Inclusion criteria**

- English language
- Only peer-reviewed scholarly articles and conference papers (In this study, we limited the inclusion criteria to peer-reviewed journal articles and full conference papers to ensure a high level of quality and academic rigor. We excluded book chapters, dissertations, and conference abstracts as they often lack the comprehensive detail, consistency, and peer-review standards needed for robust analysis, thereby maintaining the relevance and feasibility of our scoping review.)
- Subject matter
  AgeTech-related technology standards or guidelines (using the definition outlined in the third paragraph of the Background section)
- No restrictions on publication date

**Exclusion criteria**

- Non-English
- Article types
  Book chapters
  Dissertations
  Conference abstracts
  Reports
- Subject matter
  Standards or guidelines that do not emphasize age-related technology
  Standards or guidelines intended for devices specifically aimed at children aged <18 years
  Standards or guidelines intended for medical devices delivering clinical health care services within a clinical setting that require professional or medical expertise for use, monitoring, and interpretation (includes all class II, III, and IV medical devices and some class I medical devices if they are intended for medical use)
  Documents addressing frameworks and models

### Data Extraction

Data were extracted into an Excel worksheet with a variety of categories that systematically captured information relevant to the studies’ objectives and research questions. Details extracted from documents included author and year, journal or conference name and scope, country, type of study, the document’s type, aim of study, AgeTech type, developer of the standard or guideline, the old or new feature of the standard or guideline (the meaning of this variable is the response to the question: was the standard or guideline developed for the first time in this study, or was it developed previously?), methodology of standard or guideline development, target stakeholders of the standard or guideline (the meaning of this variable is the response to the question: who is going to use these standards and guidelines in AgeTech development?), characteristics of older adults as end users of AgeTech, main aspects of the standards or guidelines, results of the evaluation of the developed standard or guideline, important conclusions of the study, main limitations of the study, and gap of the study and recommendations for future studies.

### Data Analysis and Presentation of Results

Both quantitative and qualitative methods were used for data analysis. We used descriptive methods to analyze quantitative variables and then conducted a thematic analysis on data from 3 qualitative variables.

For quantitative analysis, extracted data were imported into SPSS (version 26; IBM Corp), and basic descriptive statistics were calculated, representing information on the publication year, country, journal or conference name, scope of the journal or conference, type of study, the document’s type, the old or new feature of the standard or guideline, AgeTech type, developer of the standard or guideline, target stakeholders of the standard or guideline, characteristics of older adults as end users of AgeTech, and main aspects of the standard or guideline.

For qualitative analysis, we used thematic analysis to examine 3 qualitative variables: the aim of the study, the main limitations of study, and gap of the study and recommendations for future studies. Thematic analysis is a widely recognized method in both scoping and systematic reviews and has been applied in numerous studies to provide a deeper understanding of qualitative data [23-26]. Data from each variable were individually imported into NVivo (version 14; QSR International). Two coders conducted a thematic analysis following the guidelines established by Thomas and Harden [27]. Thematic analysis was conducted in 3 stages: free coding of primary study findings, organization of these “free codes” into related areas to form “descriptive” themes, and the development of “analytical'” themes.

## Results

### Overview

**Figure 1:**
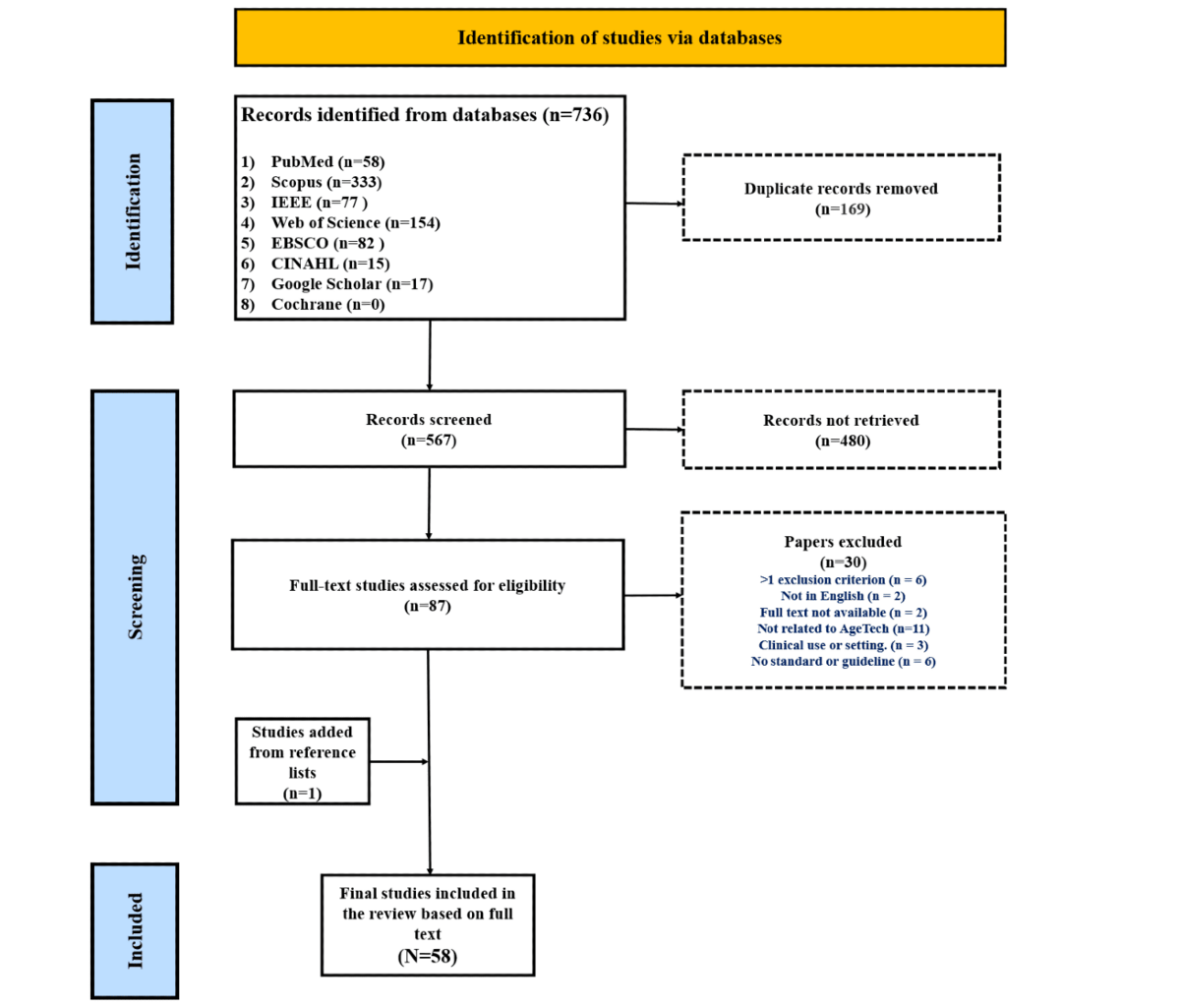
Inclusion flowchart for peer-reviewed articles, based on the PRISMA (Preferred Reporting Items for Systematic Reviews and Meta-Analyses) guidelines.

We selected 58 research papers [19,28-84] from an initial pool of 736 studies identified through the database search. We organized the information extracted from the 58 included documents into Multimedia Appendices 1-3. Figure 1 outlines the full process based on PRISMA (Preferred Reporting Items for Systematic Reviews and Meta-Analyses) guidelines.

Multimedia Appendix 1 [19,28-84] presents a summary of the studies, detailing 6 variables: publication year, country, journal or conference name, scope of the journal or conference, type of study, and document’s type. Multimedia Appendix 2 [19,28-84] compiles variables that most of them analyzed through quantitative analysis in the subsequent stage, featuring 8 columns: the old or new feature of the standard or guideline, AgeTech type, developer of the standard or guideline, methodology of standard or guideline development, target stakeholders of the standard or guideline, characteristics of older adults as end users of AgeTech, main aspects of the standard or guideline, and results of the evaluation of the developed standard or guideline. Finally, Multimedia Appendix 3 [19,28-84] summarizes variables that 3 of them analyzed using qualitative analysis in the next stage, including aim of the study, important conclusions, main limitations of the study, and gaps in the study, along with recommendations for future research. In the following sections, we will present the analysis results for Multimedia Appendices 1-3.

### Characteristics of the Study and Literature Distribution

Conference papers made up the majority of publications at 59% (34/58), while journal papers accounted for 41% (24/58). The field of computer science accounted for 69% (40/58). Other areas included health sciences (5/58, 9%), management and business sciences (2/58, 3%), multidisciplinary studies (9/58, 15%), and various other fields (2/58, 3%). Most of the publications were original research (46/58, 79%), while review articles constituted 21% (12/58) of the total. The research methods varied, with qualitative methods being the most common (37/58, 64%). Review articles also incorporated a review methodology (12/58, 21%), while quantitative methods (6/58, 10%) and mixed methods (3/58, 5%) were less commonly used (Multimedia Appendix 1). Table 1 provides a summary of the retrieved publications’ characteristics.

**Figure 2:**
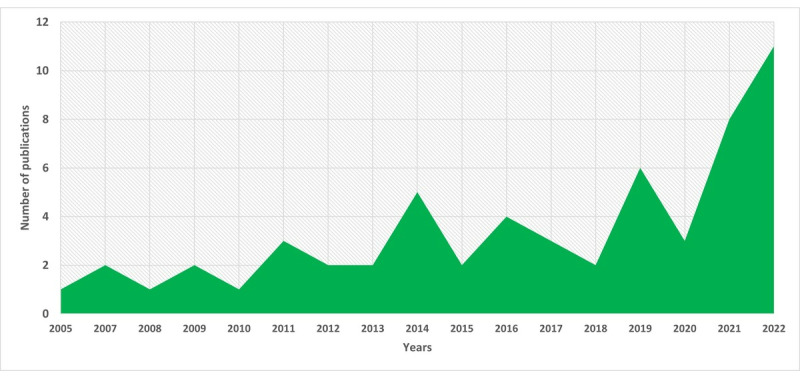
Distribution of documents by year.

Most of the papers (28/58, 48%) were published from 2019, as illustrated in Figure 2.

The United States was the leading source of publications (8/58, 14%), while Germany and Portugal each contributed to 9% (5/58) of the publications (Table 2).

**Table 1:** Summary of the retrieved publications’ characteristics (N=58).

		Publications, n (%)
**Document type**		
	Conference paper	34 (59)
	Journal paper	24 (41)
**Scope of journals or conferences**		
	Computer sciences	40 (69)
	Health sciences	5 (9)
	Management and business sciences	2 (3)
	Multidisciplinary	9 (15)
	Other	2 (3)
**Research type**		
	Original	46 (79)
	Review	12 (21)
**Research methods**		
	Review	12 (21)
	Qualitative	37 (64)
	Quantitative	6 (10)
	Mixed	3 (5)

**Table 2:** Source of publications by country (N=58).

	Publications, n (%)
Australia	2 (3)
Austria	1 (2)
Belgium	3 (5)
Brazil	1 (2)
Canada	3 (5)
China	1 (2)
Denmark	1 (2)
Finland	3 (5)
France	1 (2)
Germany	5 (9)
Greece	3 (5)
India	1 (2)
Italy	2 (3)
Japan	2 (3)
Malaysia	1 (2)
Mexico	2 (3)
The Netherlands	3 (5)
Poland	1 (2)
Portugal	5 (9)
Saudi Arabia	1 (2)
South Korea	1 (2)
Spain	1 (2)
Sweden	1 (2)
Switzerland	1 (2)
Taiwan	1 (2)
Thailand	1 (2)
United Kingdom	2 (3)
United States	8 (14)

### Findings From the Descriptive Quantitative Analysis

The analysis of the data included in Multimedia Appendix 2, regarding the status of standards or guidelines as “new” or “old,” indicated that a majority, 55% (32/58) instances, were classified as “new.” In contrast, 24% (14/58) of the instances were labeled as “N/A” (not applicable), indicating that the development status was either not applicable or not specified. The “old” category, signifying previously established standards or guidelines, accounted for 21% (12/58) of the instances.

In categorizing AgeTech types, we adopted a classification framework developed by AGE-WELL [10,85]. This framework includes 9 categories: “supportive homes and communities,” “health care and health service delivery,” “autonomy and independence,” “cognitive health and dementia,” “mobility and transportation,” “healthy lifestyles and wellness,” “staying connected,” “financial wellness and employment,” and “other.” Two authors with expertise in health informatics independently coded the AgeTech types using predefined AGE-WELL categories, achieving a high intercoder reliability with a Cohen κ score of 0.9, indicating strong agreement. Results indicate that the most prevalent AgeTech types with corresponding standards or guidelines are associated with the “staying connected” category (which includes mobile apps, social games, social and telepresence robots, friendly caller programs, and virtual reality technology), with a frequency of 31% (18/58). The “supportive homes and communities” category (encompassing smart homes, socially assistive robots, and age-friendly communities) was the second most frequent (16/58, 28%). Table 3 illustrates these findings.

**Table 3:** Distribution of studies based on AgeTech type (N=58).

	Studies, n (%)
Supportive homes and communities	16 (28)
Health care and health service delivery	7 (12)
autonomy and independence	5 (9)
Cognitive health and dementia	2 (3)
Mobility and transportation	1 (2)
Healthy lifestyles and wellness	4 (7)
Staying connected	18 (31)
Financial wellness and employment	1 (2)
Other	4 (7)

A substantially finding from the analysis pertains to the primary focus of the standards or guidelines mentioned in the selected publications. For clarity, we organized these into 11 categories. The results show that “design and development” is the most addressed aspect in AgeTech standards or guidelines, accounting for 36% (21/58) of the instances. “Usability and user experience” is the second most frequent area (11/58, 19%). In contrast, aspects receiving the least attention in AgeTech standards or guidelines in the academic literature were “ethics,” “integration and interoperability,” “accessibility,” and “acceptance or adoption,” each accounting for 3% (2/58) of the instances, and “privacy and security” and “data quality,” each accounting for 2% (1/58) of the instances. These findings are presented in Table 4. It should be noted that in this classification “design and development” encompasses all technical aspects that are important in technology design. We could classify “usability and user experience” under the “design and development” category. However, we decided not to do so because of the significance of “usability and user experience” in technology design as well as the explicit focus of studies on this aspect. Therefore, we preferred to separate them from the broader “design and development” aspect. Furthermore, it should be noted that “not applicable” refers to documents where we could not identify specific aspects related to the standards or guidelines they addressed. In contrast, “others” refers to aspects that did not fit into these categories.

The most frequently targeted stakeholders for whom standards or guidelines were developed were “designers and developers,” constituting 33% (19/58). Multiple stakeholders were the second most frequently mentioned, representing 28% (16/58) of the occurrences. Table 5 illustrates this discovery.

“General older adult population” was the largest group of end users for AgeTech, accounting for 71% (41/58). This classification was applied to end users of AgeTech in cases where the study did not define or describe the specific characteristics of older adults but only mentioned older adults or older population. Table 6 presents these results.

**Table 4:** The focus of guidelines or standards in studies based on frequencies (N=58).

	Studies, n (%)
Not applicable	9 (16)
Usability and user experience	11 (19)
Ethics	2 (3)
Accessibility	2 (3)
Data quality	1 (2)
Design and development	21 (36)
Cultural competence	3 (5)
Acceptance or adoption	2 (3)
Privacy and security	1 (2)
Integration and interoperability	2 (3)
Other	4 (7)

**Table 5:** Frequency of target stakeholders of guidelines or standards (N=58).

	Studies, n (%)
Not applicable	12 (21)
Various stakeholders	16 (28)
Designers and developers or companies	19 (33)
Health care providers	1 (2)
Older adults	7 (12)
Researchers	2 (3)
Other	1 (2)

**Table 6:** Frequency of end users of AgeTech demographics (N=58).

	Studies, n (%)
General older population	41 (71)
Older adults with cognitive problems such as dementia	5 (9)
Older adults who require residential care	5 (9)
Older adults with chronic conditions	2 (3)
Other	5 (9)

### Findings From the Thematic Analysis of Qualitative Data

The thematic analysis, which focused on 3 qualitative variables: “the aim of the study,” “the main limitations,” and “recommendations for future research” (summarized in Table 7). This can also be seen in Multimedia Appendix 3.

In the thematic analysis for the “aim of the study,” certain themes, namely, “usability studies and design guidelines,” “assistive technologies for aging,” and “human-centered design and cultural considerations,” were the most frequently referenced in the identified literature. Conversely, critical aspects such as privacy and security, ethical considerations, accessibility considerations, integration and interoperability, user adoption, and data quality had the fewest references in the literature. This finding suggests a noteworthy emphasis within the academic literature on specific themes related to the “aims of the study” in the field of AgeTech. The recurrent references to these 3 main themes indicate a predominant focus on practical and user-centric aspects of technology development, particularly those tailored to aging populations. In contrast, the fewer references to the mentioned aspects indicate potential gaps in the current research landscape. This could imply that the existing literature may not adequately address these crucial dimensions, and there might be a need for more research and attention in these areas to ensure a comprehensive and ethically sound approach to AgeTech development. The findings underscore the importance of a balanced and holistic perspective when conducting research in AgeTech, urging scholars and practitioners to broaden their focus beyond usability and design guidelines to also address ethical, privacy, and accessibility considerations essential for the responsible and inclusive development of technologies for aging populations. In addition, these results indicate that assistive technologies and ambient assisted living (AAL) technologies constitute a significant portion of references in the AgeTech domain. It also suggests that one of the most crucial subsections within the AgeTech domain is associated with solutions related to AAL. These results align with the findings derived from our quantitative analysis. In addition, regarding the thematic analysis for the “aim of the study,” we can summarize all themes into 3 general domains: “inclusive design and accessibility,” “technology-enabled health and well-being,” and “ethics, security, and digital governance.” Textbox 3 illustrates this summary of themes for the aim of the study.

**Table 7:** Summary of results from the thematic analysis of qualitative data for 3 qualitative variables.

Goal and key emergent themes			Publications defining this theme, n		A short explanation of the theme		Single quotes from literature
**Aim of study**							
	Usability studies and design guidelines	34		Numerous studies aimed to enhance the usability of mobile devices for older adults through the development of comprehensive design guidelines. For instance, 1 study emphasized the creation of a robust set of design guidelines based on 4 design strategies to ensure the usability of mobile devices for older users.		“The purpose of this research study was to develop a robust, integrative set of design guidelines based on the four design strategies to ensure usability of mobile devices by older adults.” [35]	
	Assistive technologies for aging	14		A significant theme emerged around the exploration of assistive technologies for aging individuals. Studies within this theme proposed procedures and guidelines to support further research projects, contributing to internal quality control for testing assistive technologies in real-life settings, such as living laboratories at home.		“The procedure is suggested as a guideline to support further research projects and to contribute to an internal quality control of testing involving people 65+ testing assistive technologies in Living Labs at home.” [59]	
	Human-centered design and cultural considerations	12		This theme delved into the incorporation of culturally competent assistive behaviors in robots. Studies discussed how guidelines could be encoded in robots to effectively adjust their actions, gestures, and communication to align with diverse cultural contexts.		“This paper discussed how guidelines describing culturally competent assistive behaviors can be encoded in a robot to effectively tune its actions, gestures and words.” [39]	
	AAL^a^	5		In this theme, researchers aimed to understand the standards and policy guidelines used by companies in creating AAL technologies. The goal was to identify gaps between available technologies, standards, and policies and what should be available for use in AAL applications.		“The aim of this study was to understand the standards and policy guidelines that companies use in the creation of AAL technologies and to highlight the gap between available technologies, standards, and policies and what should be available for use.” [19]	
	Cognitive stimulation and health software	7		This theme focused on gaining insight into the mistakes made by individuals with mild dementia during wayfinding on independent walks. The objective was to understand cognitive stimulation and the impact of health software in addressing these challenges.		“The goal of our study is to gain insight into frequently made mistakes that people with mild dementia make in wayfinding, while taking an independent walk.” [28]	
	Digital impact on older adults	6		This theme explored the global survey on aging-inclusive digital economy and related standards. The studies aimed to clarify the influence and challenges of the digital economy on the older population, emphasizing the expectations and demands of building an aging-inclusive digital economy.		“This paper introduces the global survey on ageing-inclusive digital economy and related standards conducted in early 2021, which aimed to clarify the influence and challenges of the digital economy on the elderly, and the expectations and demands of building an ageing-inclusive digital economy.” [26]	
	Ethical guidelines and considerations	5		This theme addresses ethics, wherein studies reviewed literature on ethics and home monitoring technology. They proposed ethical models for technology development, discussed issues for reviewers to consider, and recommended ethical guidelines to direct the research and implementation process.		“This paper aims to 1) review the relevant literature specific to ethics and home monitoring technology, 2) present an ethical model for technology development, 3) raise pertinent issues for reviewers to consider in assessing applications, 4) discuss strategies to address IRB concerns, and 5) recommend ethical guidelines to direct the research and implementation process.” [54]	
	Security considerations	2		This theme focused on analyzing the security requirements and challenges of eHealth IoT^b^ applications. Studies proposed complete architectures to address security concerns in eHealth IoT applications.		“Analyze the security requirements and challenges of e-Health Internet of Things (IoT) applications and propose a complete architecture to address them.” [63]	
	Telehealth and remote monitoring	3		This theme aimed at developing telehealth principles and guidelines specifically tailored for older adults.		“Development of telehealth principles and guidelines for older adults.” [34]	
	Accessibility considerations	1		This theme contains 1 study that emphasized the unique accessibility issues for persons with disabilities and older adults in online communities.		“The primary goals of this article are to raise awareness of the unique issues of accessibility for persons with disabilities and older adults in online communities and to identify key considerations for future development and research.” [32]	
**Main limitations of study**							
	Scope limitations	9		In this theme, studies acknowledged limitations concerning the scope of proposed guidelines. Notably, some topics crucial to care delivery, such as HIPAA^c^ adherence, data privacy, and reimbursement, were not directly addressed. A representative quote emphasized the need for a more comprehensive approach.		“A limitation is that the proposed guidelines are not all-encompassing and certain topics important to care delivery were not directly addressed. These topics include Health Insurance and Portability Accountability Act adherence, data privacy, and reimbursement.” [34]	
	Sample size and recruitment	7		This theme highlighted the limitation associated with sample size and recruitment. Studies frequently acknowledged small sample sizes, as illustrated by 1 study that mentioned a small interviewee sample of 28 participants as a potential constraint.		“The interviewee sample was small at 28 participants.” [77]	
	Validation and bias	7		This theme emerged in studies using design guidelines primarily intended for web applications. In some cases, these guidelines may not be directly applicable to mobile platforms, and there may be bias in the interpretation of the transition from Culture Interface Design Matrix to design decisions. The quote reflects this concern.		“Aaron Marcus’s design guidelines are mainly for web, so the design guidelines may not be applicable on mobile in some cases. In addition, the transition from Culture Interface Design Matrix to design decision is interpreted by the research team. Thus, potential bias may exist.” [41]	
	Data collection and analysis limitations	4		This theme highlights studies pointing out limitations in the current approach to interface analysis, which often involve manual processes. This was recognized as time consuming due to multiple scopes and technical debt. Recommendations were made for the automation of the process to enhance efficiency, reduce costs, and maximize test coverage.		“The current approach to interface analysis involves manual analysis, which is time-consuming due to multiple scopes and technical debt. To streamline the process, automation is recommended, offering benefits like cost reduction and test coverage maximization.” [29]	
	Technology and interface limitations	4		This theme highlights the challenges of integrating nonstandardized and standardized wearable activity trackers. Although the approach provided a broad integration, there were difficulties in incorporating proprietary interfaced devices, as they were either unreadable or inaccessible.		“Although the approach provided a very broad integration of nonstandardized as well as standardized wearable activity trackers, it was challenging to integrate existing proprietary interfaced devices as they could not be read or be accessed.” [80]	
	Methodological and research design	3		In this theme, the practical circumstances associated with evaluation studies were acknowledged as potential sources of deviation from standards, leading to heterogeneity in evaluation methodologies. This theme emphasized the impact of real-world circumstances on the adherence to predefined standards in evaluation studies.		“The practical circumstances of an evaluation study can cause deviations in the standards, thus producing heterogeneity in the evaluation methodologies. [65]	
	External factors impacting the study	2		In this theme, the analysis identified external factors impacting studies in the AAL field. Despite extensive research efforts, the proliferation of AAL technologies into real-world use has not matched expectations. This limitation was attributed to various research and industry organizations active in the field.		“This review has identified a high number of research and industry organizations who are currently active within the AAL field. However, the extensive research effort has not yet led to a significant proliferation of technologies into real world usage.” [25]	
**Main study recommendations for future research**							
	Refinement and iteration of guidelines	10		This theme predominantly focused on refining and iterating guidelines for various domains, such as psychotherapy, special needs education therapy, jobs screening, and occupational therapy. The aim is to enhance intervention practices, achieve more accurate measurements through game-based approaches, and explore different types of evaluations involving psychotherapy experiments using intelligent methods.		“The proposed criteria and guidelines can be adapted to other psychotherapy domain, such as special needs education therapy, jobs screening, and occupational therapy. Furthermore, this could possibly improve the existing intervention practices by having more accurate measurement through a game-based approach. Future works might consider different type of evaluation involving psychotherapy experiments using intelligent manners.” [42]	
	Future plans for evaluation and trials	9		This theme contained studies highlighting the need for future evaluations and trials of the proposed guidelines. These included user testing, proofs of concept, experiments with developers and older adults, and other assessment methods to ensure the effectiveness of the guidelines in real-world scenarios.		“As future work, new studies will be considered to evaluate the set of guidelines (AGE 1.2.), such as: user testing, proof of concepts, experiments with developers and seniors, among others.” [27]	
	Design for aging and user-centered technologies	9		This theme emphasized the evaluation of persuasive strategies and their impact on the motivation of older adults to exercise. Long-term goals include further guideline development to assist researchers and practitioners in designing user-centered assistive persuasive technologies for and with older people.		“As future work, we plan to evaluate the effectiveness of our persuasive strategies and their influence on the elderly’s motivation to exercise. In long-term perspective, we aim to develop our guidelines further in order to help other researchers and practitioners to design user-centered assistive persuasive technologies for and together with elderly.” [45]	
	Dissemination, implementation, and engagement	8		This theme highlighted the next steps in research that involve investigating older adults’ opinions of robotic assistance, assessing the length of engagement, and exploring robot assistance for older adults with impairments. In addition, this theme suggests a focus on real-world engagement and practical applications of assistive technologies.		“The next step in this line of research would be to investigate older adults’ opinions of seeing the robot in person, length of engagement, or robot assistance for older adults with impairments.” [36]	
	Development of assistive technologies	6		This theme emphasized future plans to develop an editing tool for therapists, allowing them to customize exercises based on user preferences. Additionally, the goal is to expand mobile games to include more complex exercises related to daily living activities, enhancing the transfer of learning to real-world contexts.		“Future plans involve creating an edition tool for therapists that allows for customization of exercises to fit user preferences. The game will be developed for mobile devices and expanded with more complex exercises related to daily living activities, such as shopping, money management, and social relationships, to enhance learning transfer to real contexts.” [30]	
	Ethical awareness and AI^d^ decision-making	4		This theme contained studies recommending further exploration of the effectiveness of ethical guidelines and a comparison of methods for promoting ethical awareness in the context of AI decision-making.		“One subject for the further studies could be to study the effectiveness of the ethical guidelines or to compare different methods of promoting ethical awareness.” [31]	
	Refinement of design and data	4		This theme highlighted the need for extending the proposed data format beyond body-worn sensors to include signals from other sources, such as video cameras and ambient sensors. Further efforts were deemed necessary to enhance the applicability of the presented consensus.		“The presented consensus focused only on data recorded with body-worn sensors. The proposed data format should be applicable to other type of signals, for example, from video cameras and ambient sensors, but further efforts are needed to extend the concept.” [33]	
	Aging inclusivity	3		This theme included recommendations focusing on standards and conformity assessment activities for an aging society and the silver economy. The goal of this theme is to promote research on standardization in the silver economy, encourage stakeholder involvement, and establish a long-term dialogue mechanism.		“The paper recommends a focus on standards and conformity assessment activities for the ageing society and silver economy, discussing problems and solutions, and sharing best practices among countries, territories, and organizations. It also suggests conducting research on standardization of the silver economy, encouraging more involvement, and creating a long-term dialogue mechanism.” [26]	
	Universal accessibility of online communities	2		This theme urged researchers to contribute to creating universally accessible online communities by developing accessibility guidelines, conducting studies on barriers faced by different groups, and exploring features that provide equal access.		“Researchers can contribute to creating universally accessible online communities by developing accessibility guidelines, conducting studies on barriers faced by these groups, and exploring features that would provide equal access.” [32]	
	Developing best practices	1		These themes include a single publication that covered various topics, such as developing best practices, ethnography and co-design sessions, privacy and security in AAL technology, safety and instructions in navigational aids, and validation and user experience studies.		“The paper recommends a focus on standards and conformity assessment activities for the ageing society and silver economy, discussing problems and solutions, and sharing best practices among countries, territories, and organizations.” [26]	
	Ethnography and co-design sessions	1		These themes include a single publication that covered various topics, such as developing best practices, ethnography and co-design sessions, privacy and security in AAL technology, safety and instructions in navigational aids, and validation and user experience studies.		“An ethnography could give us a much better understanding of how well seniors would be able to use the system in real conditions. Future work should also include the perspective of the rest of the stakeholders, as they can provide a more comprehensive view on this strategic application domain.” [40]	
	Focus on standards and conformity assessment activities	1		These themes include a single publication that covered various topics, such as developing best practices, ethnography and co-design sessions, privacy and security in AAL technology, safety and instructions in navigational aids, and validation and user experience studies.		“The paper recommends a focus on standards and conformity assessment activities for the ageing society and silver economy, discussing problems and solutions, and sharing best practices among countries, territories, and organizations.” [26]	
	Privacy and security in AAL technology	1		These themes include a single publication that covered various topics, such as developing best practices, ethnography and co-design sessions, privacy and security in AAL technology, safety and instructions in navigational aids, and validation and user experience studies.		“In terms of Privacy by Design guidelines, future work could include data minimization and anonymization.” [63]	
	Safety and instructions in navigational aids	1		These themes include a single publication that covered various topics, such as developing best practices, ethnography and co-design sessions, privacy and security in AAL technology, safety and instructions in navigational aids, and validation and user experience studies.		“In further studies one could concentrate on adding safety and warning instructions to the default left and right instructions of the current navigational aids for pedestrians.” [28]	
	Validation and user experience studies	1		These themes include a single publication that covered various topics, such as developing best practices, ethnography and co-design sessions, privacy and security in AAL technology, safety and instructions in navigational aids, and validation and user experience studies.		“We found that more validation and user experience studies are required to produce better AAL systems with additional user feedback and participatory development approaches.” [25]	

^a^AAL: ambient assisted living.

^b^IoT: Internet of Things.

^c^HIPAA: Health Insurance and Portability Accountability Act.

^d^AI: artificial intelligence.

**Summary of themes for the aim of the study.**

**Inclusive design and accessibility**

- Usability studies and design guidelines
- Assistive technologies for Aging
- Human-centered design and cultural
- Accessibility considerations 

**Technology-enabled health and well-being**

- Ambient assisted living
- Cognitive stimulation and health software
- Telehealth and remote monitoring

**Ethics, security, and digital governance**

- Ethical guidelines and considerations
- Security considerations
- Digital impact on the older adults

In the thematic analysis for the “main limitations of study,” the results revealed that certain themes, including “scope limitations,” “sample size and recruitment,” and “validation and bias,” were more frequently referenced in the included documents. On the basis of this finding, 1 hypothesis could be that the limitations mentioned in the literature are likely linked to the complex nature of developing standards or guidelines. Furthermore, it would be advisable for researchers to strive to mitigate these limitations in their future studies. In addition, regarding the thematic analysis for the “main limitations of study,” we can summarize all themes into 3 general domains: “scope and applicability limitations,” “methodological limitations,” and “external and contextual factors.” Textbox 4 illustrates this summary of themes for the main limitations of the study.

In the thematic analysis of “recommendations for future research” within the main study, we found that specific themes, such as “refinement and iteration of guidelines;” “evaluation and trials of standards or guidelines;” “design for aging and user-centered technologies;” and “dissemination, implementation, and engagement” were more frequently referenced in the examined literature. These suggestions indicate a forward-thinking and comprehensive approach, addressing not only the theoretical aspects of guideline development but also emphasizing the practical facets of implementation and user engagement. In addition, regarding the thematic analysis for the “recommendations for future research,” we can summarize all themes into 6 general domains: “guideline development and refinement;” “evaluation, trials, and testing;” “user-centered and inclusive design;” “assistive and ethical technology development;” “dissemination, implementation, and real-world engagement;” and “specialized research methods.” Textbox 5 illustrates this summary of themes for the recommendations for future research.

**Summary of themes for the main limitations of study.**

**Scope and applicability limitations**

- Scope limitations
- Technology and interface limitations

**Methodological limitations**

- Sample size and recruitment
- Validation and bias
- Data collection and analysis limitations
- Methodological and research design

**External and contextual factors**

- External factors impacting the study

**Summary of themes for the recommendations for future research.**

**Guideline development and refinement**

- Refinement and iteration of guidelines
- Refinement of design and data
- Developing best practices

**Evaluation, trials, and testing**

- Future plans for evaluation and trials
- Validation and user experience studies

**User-centered and inclusive design**

- Design for aging and user-centered technologies
- Aging inclusivity
- Universal accessibility of online communities

**Assistive and ethical technology development**

- Development of assistive technologies
- Ethical awareness and artificial intelligence decision-making
- Privacy and security in ambient assisted living technology

**Dissemination, implementation, and real-world engagement**

- Dissemination, implementation, engagement
- Focus on standards and conformity assessment activities

**Specialized research methods**

- Ethnography and co-design sessions
- Safety and instructions in navigational aids

## Discussion

### Overview

In response to the global trend of an aging population and the call for increasing reliance on technology to address the challenges associated with aging, this academic literature review aims to provide a comprehensive overview of the current state of design and development guidelines and standards for AgeTech to key stakeholders in this field, such as policy makers, developers, researchers, and third parties. Our discussion is structured to reflect the findings across 3 main areas: the characteristics of the study and literature distribution, the evidence of quantitative analysis, and insights from qualitative analysis.

### Characteristics of the Study and Literature Distribution

Our analysis showed that most research studies on AgeTech standards and guidelines come from the field of computer science, particularly those focused on technical development aspects such as usability and user interface design.

In examining the characteristics of retrieved publications, the results indicated that the majority were original research. Furthermore, given that the research and development processes related to standards or guidelines tend to be qualitative in nature, this study highlighted the extensive use of qualitative methods in most publications. This finding is consistent with both previous research and our predictions.

Examining the distribution of publications over the years, there has been an increased focus on research in standards or guidelines in the AgeTech and AAL domains, particularly in the years from 2019. This trend is influenced by the growing development of AgeTech and AAL. In addition, in examining the distribution of publications by country, it is observed that scientists from the United States and several European countries, including Germany, Portugal, the Netherlands, Belgium, Finland, and Canada, contributed more publications in this domain. This finding may be associated with the varying levels of technological development and aging challenges present in these countries. It is also possible that these countries view standards and guidelines as a means to better frame AgeTech development, thereby facilitating adoption. It may also be due to the availability of AgeTech standards that are more specific to those regions.

### Evidence of Quantitative and Qualitative Analysis

Regarding the findings on the type of AgeTech referenced, the results revealed that the most frequently referenced standards and guidelines were associated with the “staying connected” category, encompassing mobile apps, social games, social and telepresence robots, friendly caller programs, and virtual reality technology, accounting for 31% (18/58) of the instances. Following closely, the “supportive homes and communities” domain, covering smart homes, socially assistive robots, and age-friendly communities, accounted for 28% (16/58) of the instances. The results also indicated a lack of standards and guidelines in existing knowledge within the academic literature in certain AgeTech domains or types such as “mobility and transportation” (including smart wheelchairs, autonomous vehicles, and transportation service mobile apps), “financial wellness and employment” (encompassing financial mobile apps, cybersecurity enhancement programs, technology-based vocational programs, and workplace accommodations), “cognitive health and dementia” (covering diagnostic and predictive tools, wandering detection, and locator and GPS tracking devices), and “health care and health service delivery” (involving nanotechnologies, wearable health technologies, telemedicine, and medication management systems). The lack of standards and guidelines in the academic literature in these specific AgeTech categories may be attributed to 2 factors. The first could be the limited development in these areas of AgeTech, and the second could be the lack of attention to creating standards or guidelines in these specific AgeTech domains. In line with the first reason, a scoping review by Bergschöld et al [11] published in 2024, which included 344 documents exploring the characteristics and range of reviews about technologies for aging in place, found that assistive technologies were the most frequently discussed AgeTech type. This finding probably corresponds with the results of our own study, where AgeTech related to assistive technologies emerged as the second most prevalent category.

A significant finding revolves around the primary aspects of AgeTech standards and guidelines: “design and development” along with “usability and user experience” emerges as the predominant focus, underscoring the industry’s emphasis (or literature’s focus) on these domains. In simpler terms, most efforts are directed toward developing or addressing standards and guidelines in these specific areas of AgeTech. Conversely, areas such as “privacy and security,” “data quality,” “ethics,” “integration and interoperability,” “accessibility,” and “acceptance or adoption” receive limited attention, indicating potential gaps in the current landscape of AgeTech standards and guidelines. In light of these findings, there is a need for increased attention to developing or addressing standards and guidelines in these domains. While it is important to clarify that the findings relate to the use of standards and guidelines in academic literature, this does not necessarily signify an absence of standards or guidelines but rather a lack of their documented use in these publications. Furthermore, it is plausible that they are being used in the industry during the development of AgeTech, but their implementation might not be documented in the published literature. In their study, Memon et al [28] conducted a literature survey to explore state-of-the-art AAL frameworks, systems, and platforms, aiming to identify essential aspects and investigate critical issues from various perspectives including design, technology, quality-of-service, and user experience. The main aspects identified in AAL systems include medical device interoperability and integration; AAL system architectures, security, privacy, and data protection; design and development methodologies for AAL systems and services; frameworks and open solutions; quality attributes such as usability, accuracy, dependability, availability, reliability, technology standards, and specifications; and user experience as well as miscellaneous research in AAL systems and reviews or surveys in AAL. These findings offer valuable insights for this study in identifying the main aspects within the ALL and AgeTech domains. It is important to emphasize that a significant area of focus for researchers is the development of guidelines for designing user interfaces and improving user experience specifically for older adults. Numerous studies and systematic reviews have focused on this domain [38,41,54,71,75,81].

Concerning the frequency of stakeholders for whom standards or guidelines were developed, it appears that due to the primary focus on “design and development” along with “usability and user experience,” it is logical that the most frequently targeted stakeholders were “designers and developers,” accounting for 33% (19/58) of the instances. Furthermore, multiple stakeholders were mentioned in 28% (16/58) of the documents.

Regarding the demographic classification of adults targeted by AgeTech standards or guidelines, the results indicate that the category “general older adult population” is the most prevalent, comprising 71% (41/58) of the studies. Other older adult categories, such as “older adults living with chronic conditions,” “older adults living in residential care,” and “older adults living with cognitive impairment,” have lower frequencies. Although these results align with the early stages of AgeTech standards or guidelines development, it is advisable for scientists, standards development organizations, or associations to focus more on specific groups or populations of older adults in the future, such as those with chronic diseases, aging in place and residential care, cognitive health, and dementia. Each specific group has distinct needs, requirements, and conditions, necessitating the development of tailored standards or guidelines. It is noteworthy that the results of Bergschöld et al [11] are consistent with our study. They reported that in most review studies on AgeTech, the general population of older adults was the most frequently mentioned type of population. This finding aligns with the results of our study.

### Limitations

First and foremost, it is essential to emphasize the importance of conducting both a gray literature review and an academic literature review to comprehensively assess the current state of standards and guidelines in the AgeTech field and identify any critical gaps. To clarify, in this project, “gray literature” specifically refers to the collection of relevant practical standards and guidelines for AgeTech design and development, typically published by standardization organizations or other reputable institutions. The gray literature review is currently underway, and the findings will be presented in a subsequent publication by our research team. In addition, despite the robust methodological aspects of the scoping review design, certain limitations should be acknowledged. While we collaborated with an information specialist and conducted pilot tests with various terms to optimize the comprehensiveness of our search strategy, and searched multiple databases using relevant keywords, it is possible that not all pertinent search terms have been included. In addition, during the search process, we only considered English-language articles. Despite our efforts, which included manual searches and consultations with experts, there may still be missing documents. Hence, it is essential to acknowledge the potential risk of overlooking relevant articles.

### Conclusions

This review aimed to comprehensively outline the current state of standards and guidelines used in AgeTech design and development as reported in academic literature. Its primary focus was to explore existing knowledge and identify key gaps in AgeTech guidelines and standards. Using a scoping review and thematic analysis, we evaluated 58 academic sources using both quantitative and qualitative methods. Our primary finding emphasizes the predominant focus on “design and development” and “usability and user experience” within AgeTech standards and guidelines, reflecting the industry’s concentrated efforts in these domains. Conversely, areas such as “privacy and security,” “data quality,” “ethics,” “integration and interoperability,” “accessibility,” and “acceptance or adoption” receive limited attention, revealing potential gaps in the use and implementation of standards and guidelines across the academic landscape. Furthermore, the study highlights significant references to the “staying connected” and “supportive homes and communities” categories within AgeTech types, whereas categories such as “mobility and transportation,” “financial wellness and employment,” “cognitive health and dementia,” and “health care and health service delivery” lack sufficient standards and guidelines in academic literature. Moreover, the study highlights the notable presence of assistive technologies and AAL technologies in AgeTech, underscoring the prevalence of these solutions within the field. These insights are valuable for stakeholders, including AgeTech innovators, policy makers, health and social care providers, researchers, and experts by experience, as they guide efforts toward priority areas within AgeTech.

## Appendix

Multimedia Appendix 1 Overview of study characteristics.

Multimedia Appendix 2 Raw data and key variables for quantitative analysis.

Multimedia Appendix 3 Raw data and key variables for qualitative analysis.

Multimedia Appendix 4 Preferred Reporting Items for Systematic reviews and Meta-Analyses extension for Scoping Reviews (PRISMA-ScR) Checklist.

## Abbreviations

AAL: ambient assisted living
PRISMA: Preferred Reporting Items for Systematic Reviews and Meta-Analyses
PRISMA-ScR: Preferred Reporting Items for Systematic Reviews and Meta-Analyses Extension for Scoping Reviews
